# Normal endothelial but impaired arterial development in MAP-Kinase activated protein kinase 2 (MK2) deficient mice

**DOI:** 10.1186/s13221-016-0038-2

**Published:** 2016-10-21

**Authors:** L. Christian Napp, Olga Jabs, Anna Höckelmann, Jochen Dutzmann, Piyush R. Kapopara, Daniel G. Sedding, Matthias Gaestel, Johann Bauersachs, Udo Bavendiek

**Affiliations:** 1 Department of Cardiology and Angiology, Hannover Medical School, Carl-Neuberg-Str. 1, 30625 Hannover, Germany; 2 Department of Biochemistry, Hannover Medical School, Hannover, Germany

**Keywords:** Sprouting, Branching, Angiogenesis, Arteriogenesis, MAP-Kinase, MK2

## Abstract

Angiogenesis is a fundamental process during development and disease, and many details of the underlying molecular and cellular mechanisms are incompletely understood. Mitogen-activated protein kinase (MAPK)-activated protein kinase 2 (MK2), a major downstream target of p38 MAPK, has recently been identified as a regulator of Interleukin 1β dependent angiogenesis in vivo, and in vitro data suggest a role of MK2 for VEGF-dependent angiogenic processes in endothelial cells. We thus hypothesized that MK2 plays a role during physiological vascular development in vivo.

Vascular development was investigated in the retina of MK2-deficient mice. Retinal angiogenesis such as sprouting, branching and pruning was unchanged in MK2^-/-^ mice compared to wildtype littermates. Early arterial development was also comparable between genotypes. However, with further expansion of vascular smooth muscle cells (SMC) during maturation of the arterial network at later time points, the number of arterial branch points was significantly lower in MK2^-/-^ mice, resulting in a reduced total arterial area in adult mice. Isolated aortic smooth muscle cells from MK2^-/-^ mice showed a more dedifferentiated phenotype in vitro and downregulation of central SMC marker genes, consistent with the known impaired migration of MK2^-/-^ SMC.

In conclusion, MK2 is not required for physiological retinal angiogenesis. However, its loss is associated with an altered genetic profile of SMC and an impaired arterial network in adult mice, indicating a distinct and probably cell-specific role of MK2 in arteries.

**Electronic supplementary material:**

The online version of this article (doi:10.1186/s13221-016-0038-2) contains supplementary material, which is available to authorized users.

## Introduction

Vascular development in higher organisms consists of two principal mechanisms: Angiogenesis, i.e. endothelial sprouting, branching, and pruning, and subsequent remodelling of the endothelial network towards a complex 3-dimensional system of arteries, capillaries and veins [1]. During sprouting angiogenesis specialized endothelial ‘tip cells’ guide the outgrowing sprout, while adjacent endothelial ‚stalk cells’ build the trunk [2, 3]. In addition branching forms interconnections between stalks, resulting in a 3-dimensional endothelial network, which is further integrated by pruning, i.e. the selective degradation of previously formed endothelial interconnections [4]. With expansion of the endothelial network vascular smooth muscle cells (SMC) cover preformed endothelial tubes, as a core step of development of the arterial system [5].

Key components of the molecular control of angiogenesis are vascular endothelial growth factor (VEGF) as well as Notch ligands and their cognate receptors [1]. However, beyond VEGF other stimuli are potent inducers of angiogenesis under special conditions, such as Interleukin 1β (IL-1β) [6, 7]. p38 MAP-kinase (MAPK) and its downstream effector MAPK-activated protein kinase 2 (MK2) are central regulators of sprouting, migration, proliferation, and actin polymerization in several cell types [8]. MK2 has recently been identified as an essential downstream component of IL-1β induced angiogenesis in vitro and in vivo [9]. While IL-1β is a central angiogenic factor during tumour-associated or inflammatory angiogenesis [6, 7], physiological angiogenesis is mainly regulated by VEGF [1]. Importantly, MK2 is supposed to be required for VEGF-dependent signalling in vitro [10]. MK2-deficient mice are viable, fertile, do not show gross developmental defects [11] and survive until senescence (own unpublished observations). While MK2-deficient mice do not show an overt phenotype under baseline conditions, they do in different animal models under stress conditions. Atherosclerosis and associated inflammatory cell infiltration are reduced in MK2-deficient mice [12], and they develop much smaller brain infarcts after arterial occlusion than wildtype mice [13]. Of note, we have recently shown that global deficiency of MK2 prevents adverse remodelling but in turn promotes endothelial healing of the arterial wall after injury at the same time [14]. We thus reasoned that MK2 might play a role during physiological angiogenesis.

Here we analysed retinal vascular development, which is prototypically controlled by VEGF, in wildtype and MK2-deficient mice [11]. We show that retinal angiogenesis is unaffected in the absence of MK2 but arterial network phenotype is impaired, which might be attributed to a cell-specific role of MK2 in vascular SMC.

## Material and methods

### Mice

We investigated vascular development in the retina of newborn and adult mice. The study was conducted conforming to the German Law for the Protection of Animals and the NIH Guide for the Care and Use of Laboratory Animals. Ethical approval was obtained from local authorities of lower saxony (No. 04-13/1194 and §4 2016/117). Male and female global MK2^+/-^ mice [11] on a C57Bl/6 J background were mated to generate litters with wildtype (WT) and knockout (MK2 KO) offspring. Mice were born at the expected Mendelian ratio, and there were no differences in retina size in both genotypes (Additional file 1: Figure S1), consistent with previous reports which demonstrated, that there is no general developmental defect in the global absence of MK2 [11].

### Retina angiogenesis model

Retina preparation and immunofluorescence of wholemounts were performed at indicated timepoints as previously described [15, 16]. In brief, retinas were dissected from PFA fixed eyes, washed in PBS and incubated with PBS/1 % BSA/0,5 % TritonX100 for 24 h. After incubation with antibodies diluted in PBS/1 mM CaCl2/1 mM MgCl2/0.1 mM MnCl2/1%TritonX100 retinas were sequentially rinsed in PBS/0.5 % BSA/0,25 % TritonX100 and PBS and mounted on glass slides with coverslips using mounting medium (DAKO). In the case of Collagen-IV staining the primary as well as the secondary antibody were diluted in PBS/1 % BSA/0,5 % TritonX100/2 % donkey serum. The following antibodies or reagents were used: Isolectin-B4-FITC (Vector, 1:100), anti-smooth muscle actin-Cy3 (SMA, Sigma, 1:200), anti-SMMHC (MC-352, Kamiya Biomedical Company), anti-Caldesmon (D5C8D, Cell Signalling), anti-Collagen-IV (Chemicon, 1:250), and appropriate Cy3- or FITC-conjugated secondary antibodies (Dianova). Stained retinas were analysed on a confocal laser scanning microscope (Leica Inverted-2, Leica Microsystems, Heidelberg) or a fluorescence microscope (Zeiss Axiovert). Quantitative analyses were done with Axiovision (Zeiss, Goettingen) and ImageJ software (NIH, Bethesda) as previously described [15, 16]. Arteries were identified in the murine retina based on vascular morphology and SMA expression of the vascular wall. Arteries identified by morphology also expressed the SMC marker gene caldesmon (Additional file 2: Figure S2). All vessels expressing SMMHC also co-expressed SMA, but not all SMA-expressing vessels co-expressed SMMHC during arterial expansion (Additional file 2: Figure S2), suggesting that SMA was still the more sensitive marker to identify SMC in the developing retina. For quantification of SMA^+^ area (Fig. 2c) wholemount fluorescence pictures of SMA-stained retinas were taken at the same fluorescence intensity on a Zeiss Axiovert microscope. Colors were then converted to black (SMA) and white (background), black pixels counted with ImageJ, and results given relative to the retinal area.

### Aortic SMC isolation

Vascular SMC were isolated from 12-week-old mice by enzymatic dispersion as described previously [14]. In brief, mice were anesthetized by intraperitoneal injection of esketamine and xylazine. Aortas were dissected, rinsed with PBS and adventitial fat was removed. Aortas were digested using a mixture of Collagenase II (CLS II, No. C2-33, Biochrom) and Elastase (No. E1250, Sigma). After washing cells were grown in dishes using DMEM (Biochrom) supplemented with 20 % fetal calf serum (PAA Laboratories) and penicillin/streptomycin.

### Immunofluorescence

Murine aortic SMC were seeded to a calculated density of 30.000 cells per 0.7 cm^2^ on chamber slides (LabTek) and grown in DMEM/20 % FCS. After gentle washing with PBS cells were fixed with Cytofix fixation buffer (BD), washed and incubated with antibodies against SMA (Cy3-conjugated, C6198, Sigma-Aldrich) and SMMHC (MC-352, Kamiya Biomedical Company), followed by washing and anti-rat Alexa 488-conjugated secondary antibody (LifeTechnologies). Nuclear staining and mounting was done with Immunoselect Antifading Mounting Medium DAPI (Dianova). Slides were analyzed on an Eclipse TE2000-S microscope (Nikon) equipped with appropriate filter blocks and image processing software (NIS Elements AR 4.20.01, Nikon).

### Quantitative real-time PCR

Total RNA isolation was performed using commercial kits (NucleoSpin RNA/Protein, Macherey-Nagel), and cDNA was synthesized using a commercial kit (High Capacity RNA-to-cDNA-Kit, Applied Biosystems). Real-time PCR was performed on a CFX96 Touch™ Real-Time PCR System (Bio-Rad Laboratories) using iQ™ SYBR® Green Supermix (Bio-Rad Laboratories). Primers were as follows: SMA forward 5′-GTC CCA GAC ATC AGG GAG TAA-3′, reverse 5′-TCG GAT ACT TCA GCG TCA GGA-3′; SMMHC forward 5′-AGA GCA AAC TCA GGA GAG GAA ACG A-3′, reverse 5′-TGA GTC CCG AGC GTC CAT TTC T-3′; mouse smoothelin forward 5′-GCG GCT CCT CCC GTC GGT CTG-3′, reverse 5′-TGC CCC AGG GTA TTT TGC TCT CAG T-3′; GAPDH forward 5′-TGC ACC ACC AAC TGC TTA GC-3′, reverse 5′-GGC ATG GAC TGT GGT CAT GAG-3′; β-actin (ACTB) forward 5′-CTT TGC AGC TCC TTC GTT G-3′, reverse 5′-CGA TGG AGG GGA ATA CAG C-3′; β-2 microglobulin (B2M) forward 5′-ATG GCT CGC TCG GTG ACC CT-3′, reverse 5′-TTC TCC GGT GGG TGG CGT GA-3′; and TATA-box binding protein (TBP) forward 5′-GAG CCA GGA CAA CTG CGT T-3′, reverse 5′-ACA GCT CCC CAC CAT GTT CTG GA-3′. All primers were designed by means of NCBI Primer-BLAST. [17] Oligos were analyzed for self- and hetero-dimers using OligoAnalyzer 3.1 [18]. Primers and expected PCR products were tested for hairpins [19] and finally purchased from Eurofins MWG Operon LLC. GAPDH, ACTB, B2M, and TBP were selected as potential reference genes using Genevestigator RefGenes (Nebion) [20]. Expression stability was measured for all selected reference genes by use of the geNorm algorithm [21]. For quantification of gene expression changes, the 2^-ΔΔCt^ method was used [22] to calculate relative fold changes normalized to the geometric mean of the three reference genes with the highest expression stability. All analyses were performed in triplicates, and either the DNA template or the reverse transcriptase was omitted for control reactions.

### Western blotting

Protein extraction was performed using commercial kits (NucleoSpin RNA/Protein, Macherey-Nagel). Western blotting was performed as previously described [23]. In brief, the cleared supernatant from lysates was run on commercial precast 4–12 % Bis-Tris gels (NuPAGE®, LifeTechnologies) and blotted onto nitrocellulose membranes (Trans-Blot®, Bio-Rad). After blocking with Amersham ECL Prime Blocking Agent (GE) blots were incubated with the primary antibody overnight at 4 °C. Primary antibodies were used against SMA (ab5694, Abcam), SMMHC (MC-352, Kamiya Biomedical Company), smoothelin (sc-28562, Santa Cruz), caldesmon (sc-15374, Santa Cruz), calponin (sc-28546, Santa Cruz), and GAPDH (5174, Cell Signaling). Proteins were detected by enhanced chemiluminescence (Pierce ECL Plus, Thermo Scientific) after labeling with a horseradish peroxidase-conjugated secondary antibody (sc-2056 or sc-2004, Santa Cruz). Densitometry was performed with ImageJ.

Graphs were plotted with Prism 6 (GraphPad). Differences between groups were evaluated with student’s *t*-test. *P* values below 0.05 were considered statistically significant. Quantitative data is provided in Additional file 3: Table S1.

## Results

**Fig. 1:**
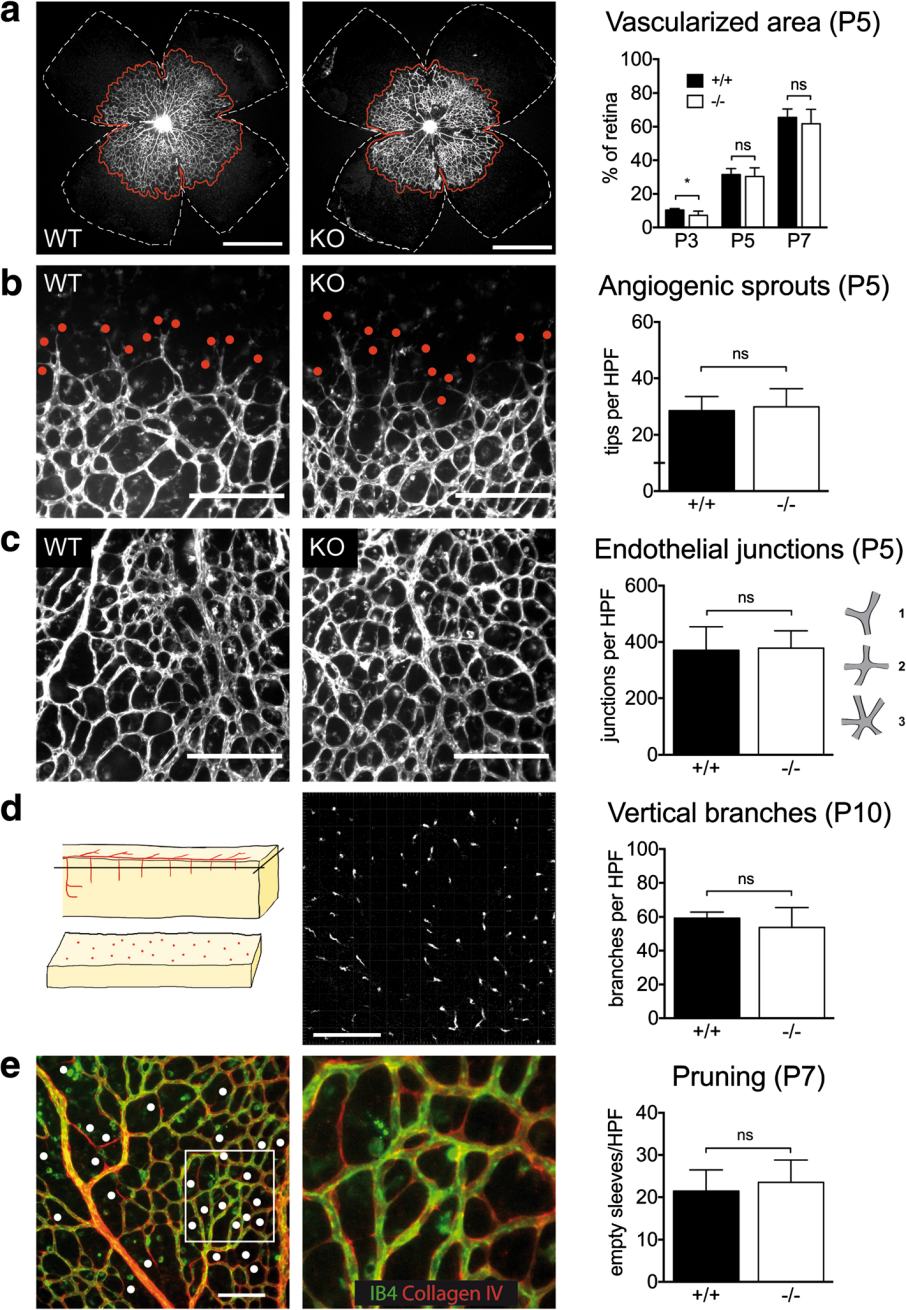
Normal Angiogenesis in MK2-deficient mice. **a** The growing endothelial plexus. Isolectin-B_4_(IB_4_)-staining, quantification of the vascularized area (*red line*) relative to whole retina area (*white line*). Scale bar: 1 mm. **b** Angiogenic sprouts (*red dots*). IB_4_-staining, quantification per high power field (HPF). Scale bar: 200 μm. **c** Endothelial junctions. IB_4_-staining. Points were assigned according to the complexity of junctions (trifurcation 1 point, quattrofurcation 2 points, pentafurcation 3 points). Scale bar: 200 μm. **d** Vertical branching. IB4-staining. Analysis was done with a confocal microscope in order to count vessels penetrating the middle layer of the retina (left). Scale bar: 200 μm. **e** Pruning. IB_4_-negative and Collagen-IV-positive sleeves were counted in HPF. Scale bar: 100 μm. P indicates postnatal day. * = *p* < 0.05, ** = *p* < 0.01

Angiogenesis was investigated in the murine retina, in which a vascular plexus develops de novo after birth [2]. In WT mice the retina was progressively grown by a primitive endothelial network, covering the inner retinal surface in a centrifugal fashion (Fig. 1a). This process was unchanged in MK2 KO mice as assessed by measuring the area covered by the endothelial cell (EC) network over time, indicating that there is no gross sprouting angiogenesis defect in MK2 KO mice. Consistently, the number of sprouts at the growing angiogenic front was comparable between genotypes at postnatal day (P) 5 (Fig. 1b). In addition, the number of branch points at P5 was unchanged (Fig. 1c), as was the number of vertical branches at P10 (Fig. 1d), demonstrating that primary development of the 3-dimensional EC network was mainly unaffected by the global absence of MK2. We next investigated pruning, which comprises retraction of EC interconnections as a central remodelling process during maturation of endothelial networks. As assessed by counting sleeves with Collagen-IV positive basal membranes lacking Isolectin-B_4_-positive EC, pruning was unchanged at P7 in both genotypes (Fig. 1e). In summary, these data indicate that MK2 is neither required for development nor for remodelling of the endothelial plexus.

**Fig. 2:**
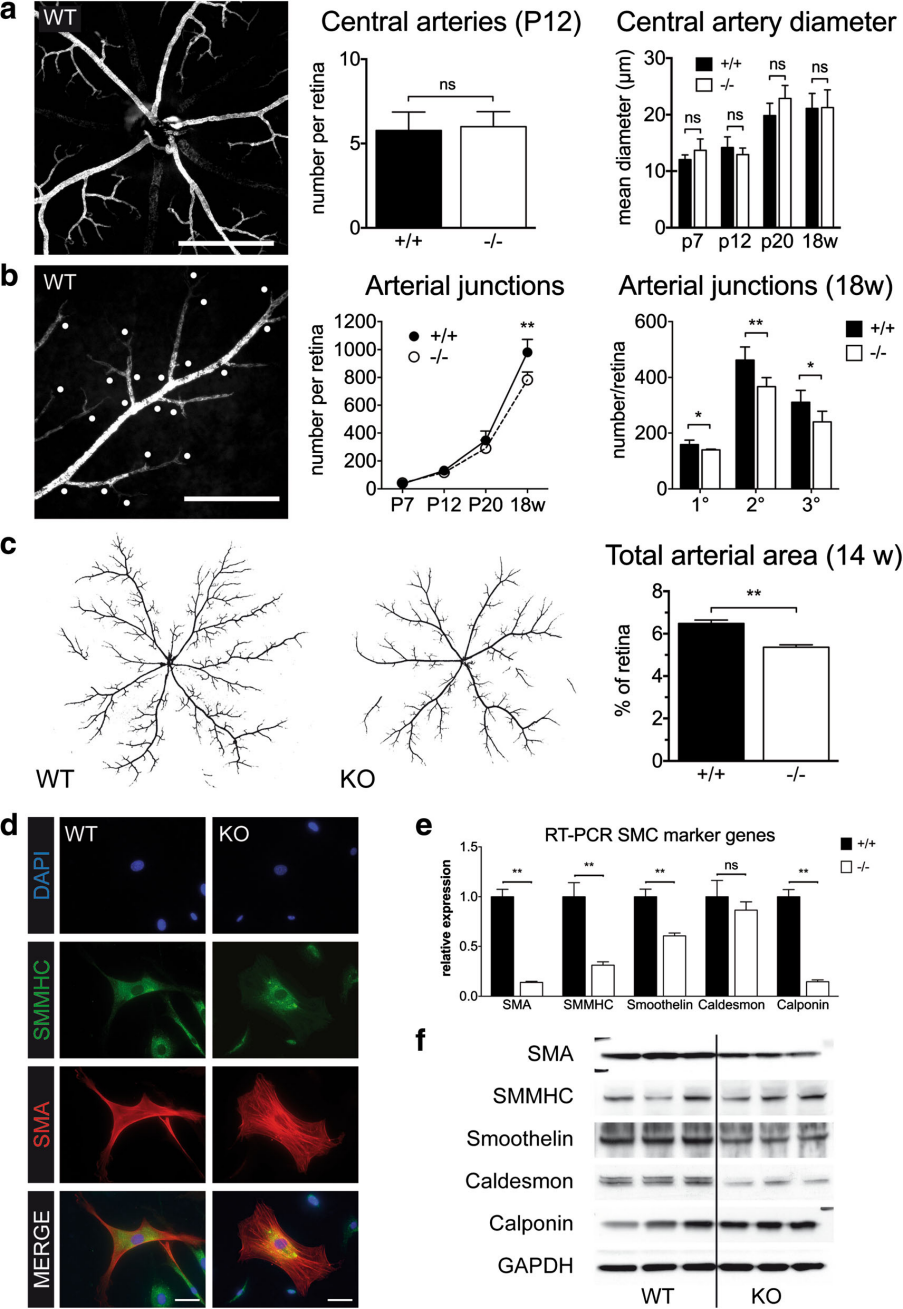
Impaired Arterial Network in MK2-deficient mice. **a** Normal number of central arteries in MK2-WT and -KO mice. SMA-staining. Scale bar: 400 μm. **b** Arterial branchpoints. KO have less branchpoints especially in higher orders of junctions. SMA-staining. Scale bar: 200 μm. **c** Reduced total arterial area in MK2-KO mice. Images were converted from SMA-stained retinas to generate a *black*-and-*white*-image. **d** Isolated SMC from WT and MK2-KO mice, immunofluorescence. Pronounced flattening of SMC in KO mice as a surrogate of dedifferentiation. Scale bar: 50 μm. **e** RT-PCR of SMC marker genes. **f** Western blotting for the same targets as in (**e**). Both **e** and **f** demonstrate reduced expression of prototypical SMC marker genes in MK2-KO mice. P indicates postnatal day, w indicates weeks. * = *p* < 0.05, ** = *p* < 0.01

After initial development a part of the EC network in the retina matures to give rise to the arterial system, whereas another part is remodelled towards a venous network, with capillaries remaining in between [24]. Regarding the previously described role of MK2 for SMC migration [25] we next investigated arterial development in the retina. The number of central arteries at P12 was comparable between WT and MK2 KO mice, as was the mean diameter of central arteries over time (Fig. 2a), indicating that the initial setup of central arteries was unaffected. In contrast, while the number of arterial (SMA^+^) branch points was comparable at early stages, from P20 on their number was progressively lower in MK2 KO mice resulting in a significant difference between genotypes in adulthood (Fig. 2b, *P* < 0.001). This reduction was apparent throughout the hierarchy of junctions, but was even more prominent in higher orders (Fig. 2b, *P* < 0.001). This indicated that impairment of “arterialization” in MK2 KO mice was more pronounced in the periphery of the arterial system, which was also overt in wholemount SMA^+^-stained retinas (Fig. 2c). As a result the total arterial area, i.e. the SMA^+^ area relative to the total retinal area, was significantly smaller in MK2 KO mice (Fig. 2c, *P* < 0.001). In summary, these data indicate that MK2 is required for normal development of the arterial system.

The phenotype observed in the retina suggested that MK2 plays a role in SMC, however compared to other tissues cells are difficult to isolate from murine retinas. We therefore isolated vascular SMC from aortas of adult WT and MK2 KO mice. Culture of aortic SMC demonstrated that MK2 KO SMC appear more dedifferentiated with a flattened morphology (Fig. 2d). Expression profiling by RT-PCR and western blotting revealed pronounced differences in the expression of prototypical SMC marker genes such as SMA, SMMHC, smoothelin, caldesmon and calponin (Fig. 2e, f and Additional file 4: Figure S3). A close-up analysis of growing retinal arteries revealed no gross differences between genotypes (Additional file 5: Figure S4) in terms of attachment of SMC or the distance between EC and SMC layers. Together with the previously published observation that SMC from MK2 KO mice show impaired migration in vitro [14, 25], our data indicate that MK2 plays an important probably cell-specific role for function or maintenance of SMC.

## Discussion

Several principle molecular and cellular components of angiogenesis have been discovered [1], but many aspects are still poorly understood. MK2 has recently been identified as a critical downstream molecule of IL-1β induced angiogenesis [9] as well as a regulator of VEGF-dependent proangiogenic processes in endothelial cells [10]. Therefore we hypothesized that MK2 plays an important role in physiological VEGF-driven angiogenesis. We assessed vascular development ex vivo in the murine retina, which is a well-established way of investigating VEGF-induced physiological angiogenesis [15, 26]. To our surprise physiological retinal angiogenesis was unaffected in MK2-deficient mice. While our previous data support a role of MK2 for enhancing angiogenesis under special conditions such as inflammation or tumour formation [9], the present data demonstrate that MK2 is not essential for normal angiogenesis at baseline. Our findings thus stand in contrast to the early report of in vitro data that MK2 is essential for VEGF-induced EC migration downstream of VEGF receptor 2 [10]. This discrepancy, however, might be explained by the fact that retinal angiogenesis depends on a complex interplay of VEGF receptors 1, 2 and 3 and not only on VEGF receptor 2 [27]. Consistently, we have previously shown that endothelial regeneration is enhanced in the absence of MK2 after carotid injury [14], further supporting that MK2 is not essentially required for endothelial growth in VEGF-dominated environments.

MK2 regulates actin-remodeling and stress fiber formation through phosphorylation of small heat shock proteins in different cell types [8]. By this, MK2 also plays an important role in migration of SMC [14, 25]. Since SMC cover preformed endothelial tubes during vascular development, we analyzed MK2-dependent development of the arterial system in the murine retina. Initial setup of central arteries was normal, but later on arterial network appearance was MK2-dependent, as reflected by a significantly lower number of arterial branch points and total arterial area in adult MK2-deficient mice. Isolated SMC from MK2-deficient mice showed a more dedifferentiated morphology paralleled by an altered expression profile of prototypical SMC marker genes, which corroborates and expands the finding that SMA expression depends on MK2 [28, 29]. The genes we found to be reduced in MK2-KO SMC are in part important for migration and contraction of SMC, and we and others have previously shown that migration of MK-deficient SMC is impaired [14, 25]. While our findings demonstrate that early arterial development does not require MK2, we found a strong arterial phenotype at later time points, associated with an altered gene profile of isolated SMC. This may either be explained by impaired migration of SMC towards the periphery of the arterial system at later stages of arterial development, or a function of MK2 for maintenance of SMC. Albeit this does not exclude a role of other cells to be involved in the observed phenotype, our results support a cell-specific role of MK2 during vascular development.

## Conclusion

In conclusion, MK2 is not required for retinal angiogenesis under baseline conditions in vivo, which is a prototype of physiological VEGF-driven angiogenesis. MK2, however, is required for marker gene expression and migration of SMC and important for normal arterial network formation. In this our study adds to our very recent finding that MK2 is essential for adaptive arteriogenesis [30]. Although many genes have been associated with arterial identity [31] with most of these also being important for angiogenesis (such as the Notch family), to our knowledge our data for the first time identify a regulator which specifically influences the arterial network in the presence of normal angiogenesis. Our data set the stage for future studies on the cell-specific role of MK2 during development and maintenance of arteries.

## Additional files

Additional file 1: Figure S1. Development of mice. A: Normal distribution of genotypes in offspring of MK2^+/-^ mice. B: Comparable retina size in MK2 WT and KO-mice. (JPG 1587 kb)
Additional file 2: Figure S2. Identification of arteries in the retina. A: Co-Staining for SMA (green) and SMMHC (red) demonstrates that SMA labels much more SMC than SMMHC. B: Staining for the SMC marker Caldesmon (red) and EC (Isolectin B4, green). Arteries and veins are readily distinguishable by the typical morphology in the retina, which was verified by SMMHC and caldesmon expression. Scale bar: 200 μm. (JPG 2011 kb)
Additional file 3: Table S1. Quantitative data analysed in the study. Measurements referring to Figs. 1 and 2. (PDF 45 kb)
Additional file 4: Figure S3. Densitometry of western blots, corresponding to Fig. 2f. (JPG 2344 kb)
Additional file 5: Figure S4. Growing artery in the retina. Staining for EC (Isolectin B4, green) and SMC (SMA, red) demonstrates close proximity of EC and SMC in the arterial wall independent of the genotype. Scale bar: 20 μm. (JPG 2939 kb)

## Abbreviations

EC: Endothelial cell
MAPK: Mitogen activated protein kinase
MK2: MAPK-activated protein kinase 2
P: Postnatal day
SMC: Smooth muscle cell
VEGF: Vascular endothelial growth factor
