# Erratum to: ‘Normal endothelial but impaired arterial development in MAP-Kinase activated protein kinase 2 (MK2) deficient mice’

**DOI:** 10.1186/s13221-016-0039-1

**Published:** 2016-12-23

**Authors:** L. Christian Napp, Olga Jabs, Anna Höckelmann, Jochen Dutzmann, Piyushkumar R. Kapopara, Daniel G. Sedding, Matthias Gaestel, Johann Bauersachs, Udo Bavendiek

**Affiliations:** 1 Department of Cardiology and Angiology, Hannover Medical School, Carl-Neuberg-Str. 1, 30625 Hannover, Germany; 2 Department of Biochemistry, Hannover Medical School, Hannover, Germany

## Erratum

Unfortunately, the original version of this article [1] contained an error. One of the author’s names was incorrect. For the author Piyush R. Kapopara, the given name should have been published as Piyushkumar instead of Piyush. This is presented correctly in the above author list and will also be updated in the original article [1].
