# Supplementary figures and images for: Normal endothelial but impaired arterial development in MAP-Kinase activated protein kinase 2 (MK2) deficient mice
Source: Vasc Cell. 2016 Oct 21;8:4. doi: 10.1186/s13221-016-0038-2 (PMC5073967; doi:10.1186/s13221-016-0038-2)

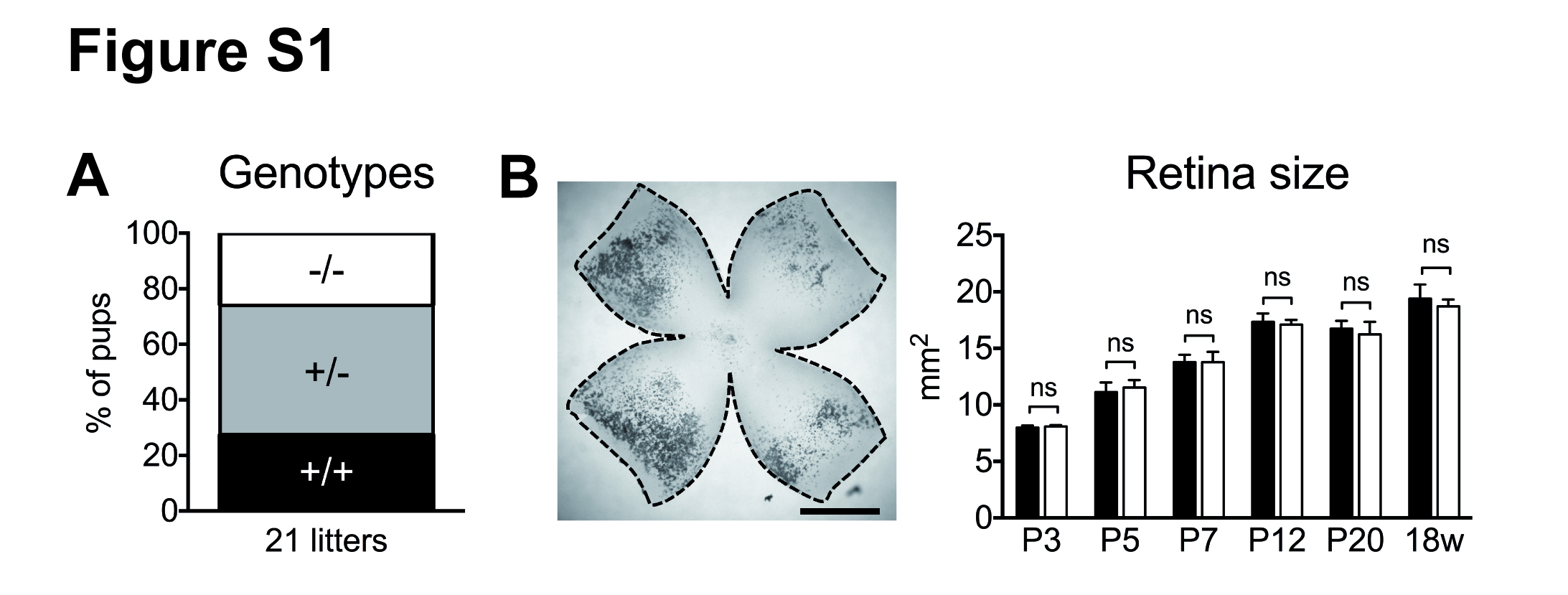

Supplement: Additional file 1: Figure S1. — Development of mice. A: Normal distribution of genotypes in offspring of MK2+/- mice. B: Comparable retina size in MK2 WT and KO-mice. (JPG 1587 kb) [file 13221_2016_38_MOESM1_ESM.jpg]

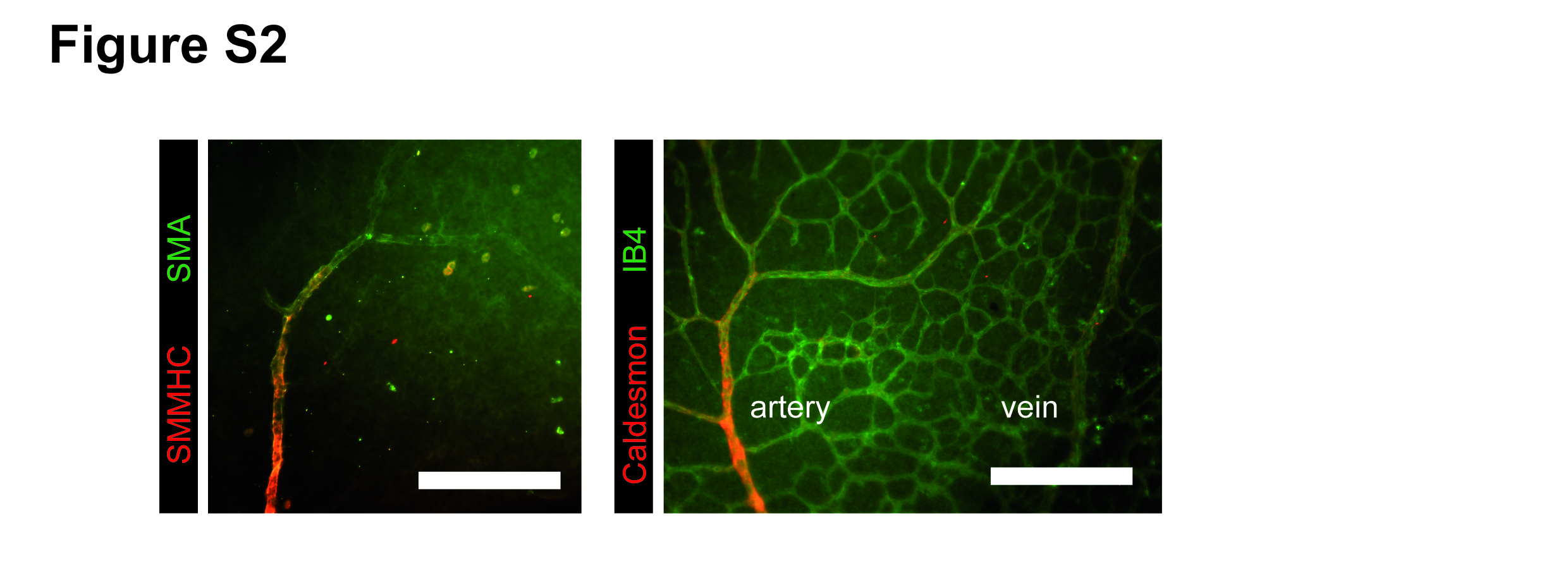

Supplement: Additional file 2: Figure S2. — Identification of arteries in the retina. A: Co-Staining for SMA (green) and SMMHC (red) demonstrates that SMA labels much more SMC than SMMHC. B: Staining for the SMC marker Caldesmon (red) and EC (Isolectin B4, green). Arteries and veins are readily distinguishable by the typical morphology in the retina, which was verified by SMMHC and caldesmon expression. Scale bar: 200 μm. (JPG 2011 kb) [file 13221_2016_38_MOESM2_ESM.jpg]

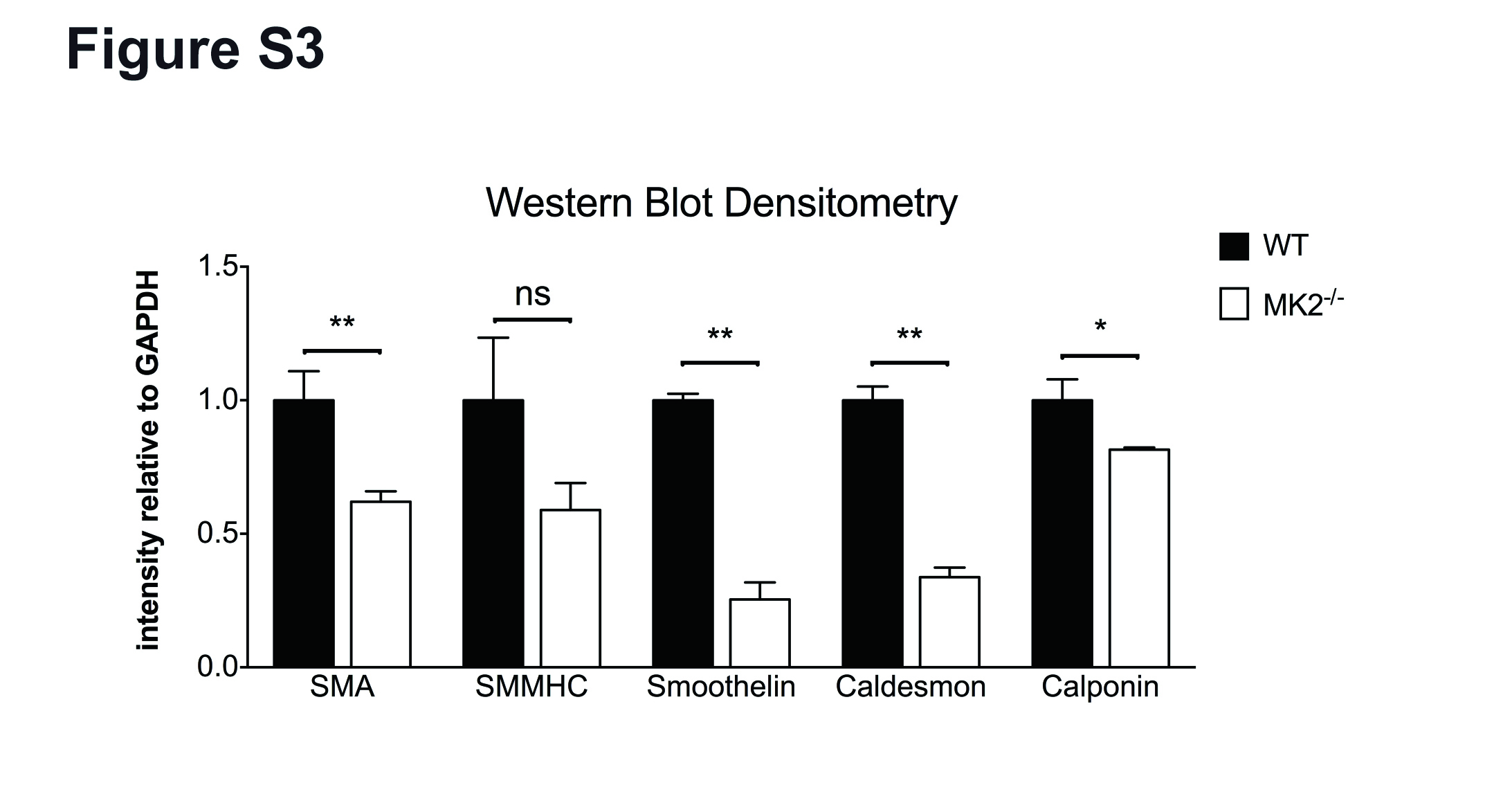

Supplement: Additional file 4: Figure S3. — Densitometry of western blots, corresponding to Fig. 2f. (JPG 2344 kb) [file 13221_2016_38_MOESM4_ESM.jpg]

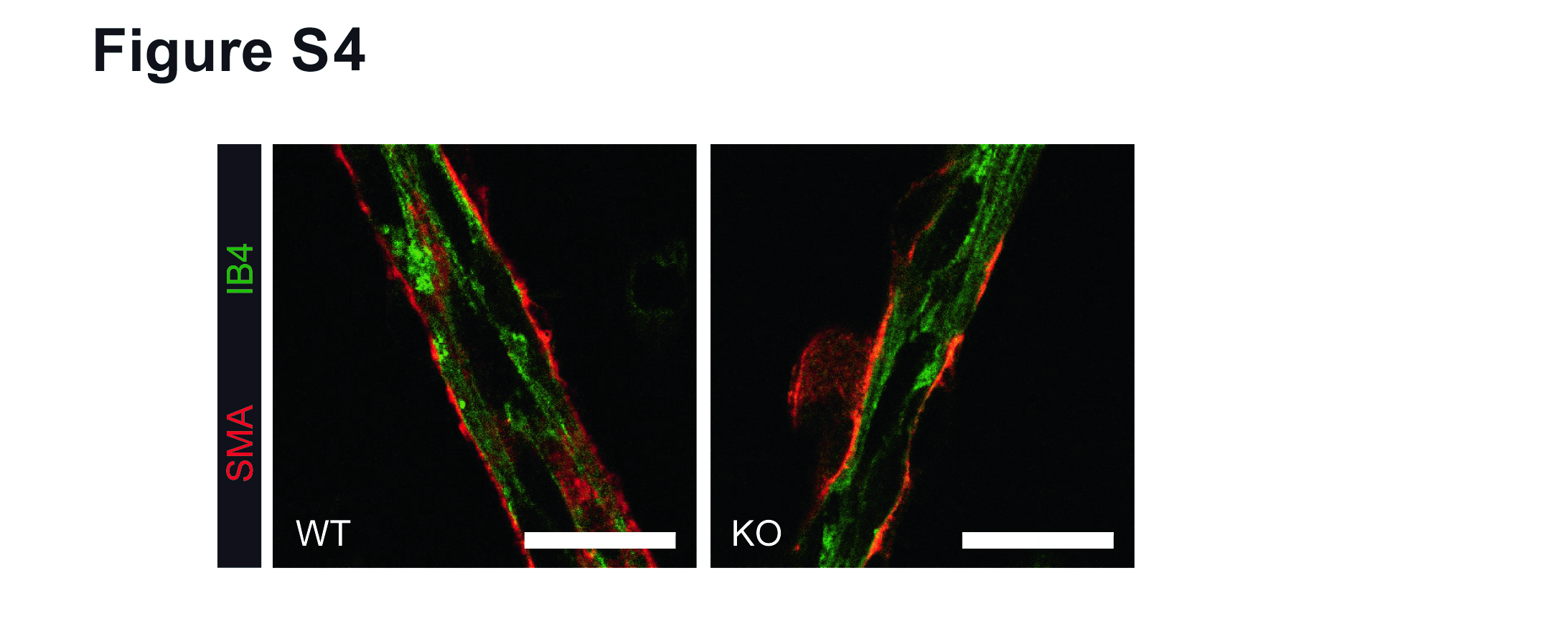

Supplement: Additional file 5: Figure S4. — Growing artery in the retina. Staining for EC (Isolectin B4, green) and SMC (SMA, red) demonstrates close proximity of EC and SMC in the arterial wall independent of the genotype. Scale bar: 20 μm. (JPG 2939 kb) [file 13221_2016_38_MOESM5_ESM.jpg]
