# Supplementary material for: Normal endothelial but impaired arterial development in MAP-Kinase activated protein kinase 2 (MK2) deficient mice
Source: Vasc Cell. 2016 Oct 21;8:4. doi: 10.1186/s13221-016-0038-2 (PMC5073967; doi:10.1186/s13221-016-0038-2)
Supplement: Additional file 3: Table S1. — Quantitative data analysed in the study. Measurements referring to Figs. 1 and 2. (PDF 45 kb) [file 13221_2016_38_MOESM3_ESM.pdf]

Table S1		Quantitative Results						
	**time**	**MK2 WT**			**MK2 KO**			**WT vs. KO**
**Angiogenesis - Fig. 1**		**N=**	**mean**	**SD**	**N=**	**mean**	**SD**	**p value**
Vascularized area - % of retina	P3	4	10,4	0,7	6	7,2	2,3	0,047
...	P5	15	31,5	3,4	11	30,4	4,9	0,514
...	P7	13	65,5	4,8	20	61,8	8,3	0,164
Angiogenic sprouts per HPF - n	P5	15	28,4	4,9	11	29,9	6,1	0,538
Endothelial junctions per HPF - n	P5	15	370,7	80,4	11	378,0	58,7	0,809
Vertical branches per HPF - n	P10	6	59,3	3,3	12	53,8	11,3	0,286
Empty Collagen IV^+-$IB4^-$ sleeves per HPF - n	P7	19	21,4	4,9	10	23,5	5,0	0,306
**Arterial development - Fig. 2**								
Central arteries - n	P12	9	5,8	1,0	6	6,0	0,8	0,686
Central arteries - mean diameter - µm	P7	5	12,1	0,7	9	13,7	1,9	0,108
...	P12	8	14,2	1,8	4	12,9	1,0	0,258
...	P20	7	19,9	1,9	4	22,9	2,0	0,056
...	18w	7	21,1	2,5	5	21,3	2,8	0,928
Arterial (SMA+) junctions per retina - n	P7	4	36,0	5,2	9	43,0	6,1	0,085
...	P12	8	131,1	21,2	6	118,0	5,6	0,196
...	P20	8	346,4	67,8	6	289,8	59,7	0,173
...	18w	7	980,6	93,2	5	782,0	57,0	0,003
Total arterial area - % of retina	14w	8	6,5	0,4	8	5,4	0,3	< 0,001
**Development - Fig. S1**			**mean**	**SD**		**mean**	**SD**	**p value**
Retina size - mm^2	P3	4	8,0	0,2	6	8,1	0,1	0,294
...	P5	15	11,1	0,8	11	11,5	0,6	0,201
...	P7	13	13,8	0,6	20	13,8	0,9	0,966
...	P12	8	17,3	0,7	6	17,1	0,4	0,499
...	P20	8	16,7	0,6	6	16,2	1,0	0,325
.	18w	7	19,4	1,2	5	18,7	0,5	0,293

HPF denotes high-power-field (20x magnification), IB4 Isolectin B4, SD standard deviation, SMA smooth-muscle actin
